# Adequacy of Antenatal Care during the COVID-19 Pandemic: Observational Study with Postpartum Women

**DOI:** 10.1055/s-0042-1755340

**Published:** 2022-11-29

**Authors:** Vanessa Martins Rosa, Betine Pinto Moehlecke Iser

**Affiliations:** 1 Universidade do Sul de Santa Catarina, Programa de Pós-Graduação em Ciências da Saúde, Palhoça, SC, Brazil

Dear Editor,

We enthusiastically read the analysis conducted in the study Adequacy of Prenatal Care During The COVID-19 Pandemic: An Observational Study with Postpartum Women, 1 published in volume 44 issue #4 of the Brazilian Journal of Gynecology and Obstetrics in 2022, and would like to bring some considerations about the topic.

According to the World Health Organization (WHO), investing in prenatal care is essential to improve one of the most important indicators of quality of life for the world population, which is maternal mortality, in which Brazil is below the rate stipulated as ideal. 2

The state of Santa Catarina and the South region, according to national data, stand out for the lowest rates of maternal mortality, better prenatal care coverage, and quality of prenatal care. 3 4 However, the few current studies analyzing the quality of prenatal care in this region differ from the data made available by the Ministry of Health (MH). 1 5 Thus, it is pertinent to emphasize that this study has great value by making a careful analysis of this assistance.

As the authors point out in the limitations of the study, the diversity of criteria used to evaluate the quality of prenatal care makes a more complete and comparative analysis of the data difficult. 1 The Brazilian government uses criteria with few variables for prenatal care adequacy, using the Kotelchuck index, 6 which takes into account only the date of prenatal care initiation and the number of appointments. Thus, the data found in national studies differ from the MH database. Between the years 2012 and 2013, Tomasi et al. 7 found in a national survey with 50,791 participants only 21% adequacy to prenatal care, while for the MH, in the year 2014, the national adequacy was 63%. Therefore, the development of robust studies that confront the findings and justify such divergence becomes pertinent.

Most studies analyze how well the current MH guidelines 8 are being met, while others analyze national and even international indexes. However, there are no uniform and well-accepted criteria for the classification of the adequacy of prenatal care; besides, there is no uniformity in the way data is collected, resulting in values with large variations and making it difficult to compare the populations studied. 9 As an example, we can cite the Northeast region of Brazil, which, according to the WHO, has a prenatal care quality index lower than the national average; 10 however, in a study conducted in Paraiba in 2018, a good quality was verified with > 80% of prenatal visits starting at the ideal time and with an adequate number of appointments, and having > 90% of the necessary exams performed. 11

According to the study by Martin et al., 1 in the year 2020, amid the COVID-19 pandemic, 35.8% of prenatal care were considered adequate, 46.8% intermediate, and 17.4% inadequate. In the previous year, according to the MH, 79.3% of prenatal care for Southern Brazil were considered adequate. This important difference is probably not only related to the pandemic, since other studies out of the pandemic period have even lower values of adequacy. 5 12 Weaknesses in assistance were found in a study from the most populous city of Santa Catarina: only 17% of the patients received proper guidance during pregnancy and less than half of the patients underwent 3 ^rd^ trimester exams (42.5%). 5

Regarding the study in question, the authors claim to use not very strict parameters to assess the quality of prenatal care, such as the week of the beginning of prenatal care being appropriate under 16 weeks; however, since 2012, the MH recommends starting before 12 weeks. 8 The procedures recommended as adequate were the performance of three tests: syphilis, HIV, and urinary tract infection in the 3 ^rd^ trimester. Additionally, since 2012, the following are recommended as essential for a quality prenatal care: immunization for hepatitis B and Diphtheria and tetanus vaccine (DT), and performance of. obstetric clinical procedures such as weight, blood pressure, edema, body mass index, uterine height, fetal heartbeat, fetal movements, fetal presentation in the appointments conducted, and laboratory tests for each trimester. In addition to the prescription of ferrous sulfate and folic acid supplements and guidance related to the prenatal period, delivery, puerperium, breastfeeding, newborn care, healthy habits, and emotional and body modifications. 8 It is assumed, therefore, that the percentage of adequacy in this study was overestimated by considering only the minimum necessary, and the impact of the pandemic on prenatal care must have been even greater than estimated.

As long as studies are conducted in a nonuniform manner, using different assessment approaches and not meeting the recommendations of the MH, 8 we will not know exactly how the healthcare of pregnant women is at the regional and national levels. Thus, a complete analysis would require the use of the different criteria available in order to allow the applicability of the results in the development of assertive improvement policies.

## Author's Reply

***Resposta do autor***

Margot Marie Martin ^1^ Roxana Knobel ^2^ Vitor Nandi ^1^ Jessica Goedert Pereira ^2^ Alberto Trapani Junior ^2^ Carla Betina Andreucci ^3,4^

^1^ Hospital Universitário Polydoro Ernani de São Thiago, Florianópolis, SC, Brazil

^2^ Universidade Federal de Santa Catarina, Florianópolis, SC, Brazil

^3^ Hospital Regional Homero de Miranda Gomes, São José, SC, Brazil

^4^ Universidade Federal de São Carlos, São Carlos, SP, Brazil

**Address for correspondence** Roxana Knobel, Departamento de Ginecologia e Obstetrícia – Bloco pedagógico – 2° andar Hospital Universitário- Rua Professora Maria Flora, Pausewang, s/n°, 88036-800, Campus Universitário, Trindade, Florianópolis, Santa Catarina, Brazil (e-mail: rknobel@gmail.com).

Dear Authors,

We appreciate the feedback on our article “Adequacy of Antenatal Care during the COVID-19 Pandemic: Observational Study with Postpartum Women”. ^1^

As you pointed out, the method applied may underestimate the percentage of people with access to adequate antenatal care, mainly because quantitative criteria were applied, and as mentioned, in a quite permissive way. However, the adopted index has a high positive predictive value on adequate antenatal care access and good levels of adjusted agreement with other antenatal assessment indices. ^2^ The same method was also used in previous publications, ^3^ ^4^ allowing comparisons. We agree that qualitative and more in-depth assessments are needed, but quantitative assessment may also reveal inequities and differences in outcomes. ^5^ In our study, for example, we found barriers to black or brown skin color women in receiving adequate antenatal care, as already described in the literature, ^3^ ^6^ a situation that was aggravated by the pandemic. ^7^

Modifying the beginning of antenatal care to ≤ 12 weeks (without modifying further criteria) in our sample, the number of postpartum people with adequate antenatal care drops from 91 to 67. Despite the highlighted limitations, our study demonstrates a decrease in access and quality of antenatal care in 2020. Instead of the decrease, an increase in adequate coverage would be expected using less rigid criteria for antenatal adequacy.

Due to scheduling difficulties, many users stopped seeking medical care or going to routine appointments in 2020, ^8^ as shown in our study. Virtual consultations were also a risk factor for inadequate antenatal care, probably because of their abrupt start during the pandemic, without former training or preparation. ^9^ Additionally, our data showed how social isolation impacted pregnant women during the 1 ^st^ year of the pandemic, when no vaccines were available, and how none of the investigated social isolation variables were related to inadequate antenatal care. ^1^

We agree with the need to standardize antenatal adequacy criteria or a series of criteria suitable for each research objective, allowing better comparisons among studies, different locations, and institutions. We also agree that qualitative approach studies are needed to assess antenatal care quality. However, we believe that this should not prevent researchers from carrying out quantitative studies. We consider that even the official public data available on the Information System on Live Births (SINASC, in the Portuguese acronym) of the Information Technology Department of the Brazilian Unified Public Healthcare System (DATASUS, in the Portuguese acronym) ^10^ may help to identify barriers to quality antenatal care access in the Brazilian population, leading to public policies to optimize obstetric care.

**Conflict of Interests**

The authors have no conflict of interests to declare.

**References**

1 Martin MM, Knobel R, Nandi V, Pereira JG, Trapani Junior A, Andreucci CB. Adequacy of antenatal care during the COVID-19 pandemic: observational study with postpartum women. Rev Bras Ginecol Obstet. 2022;44(04):398–408. Doi: 10.1055/s-0041-1741450

2 Tomasi E, Fernandes PA, Fischer T, Siqueira FCV, Silveira DS, Thumé E, et al. Qualidade da atenção pré-natal na rede básica de saúde do Brasil: indicadores e desigualdades sociais. Cad Saude Publica. 2017;33(03):e00195815. Doi: 10.1590/0102-311 × 00195815

3 Melo EC, Oliveira RR, Mathias TA. [Factors associated with the quality of prenatal care: an approach to premature birth]. Rev Esc Enferm USP. 2015;49(04):540–549. Doi: 10.1590/S0080-623420150000400002

4 Nunes JT, Gomes KR, Rodrigues MT, Mascarenhas MD. Qualidade da assistência pré-natal no Brasil: revisão de artigos publicados de 2005 a 2015. Cad Saude Colet. 2016;24(02):252–261. Doi: 10.1590/1414-462 × 201600020171

5 Rodrigues KMD, Zoldan C, Silva CBO, Santana EFM, Araujo Júnior E, Peixoto AB. Relationship between the number of prenatal care visits and the occurrence of adverse perinatal outcomes. Rev Assoc Med Bras. 2022;68(02):256–260. Doi: 10.1590/1806-9282.20211239

6 Leal MDC, Gama SGND, Pereira APE, Pacheco VE, Carmo CND, Santos RV. The color of pain: racial iniquities in prenatal care and childbirth in Brazil. Cad Saude Publica. 2017;33(33, Suppl 1)e00078816. Doi: 10.1590/0102-311 × 00078816

7 Gur RE, White LK, Waller R, Barzilay R, Moore TM, Kornfield S, et al. The disproportionate burden of the covid-19 pandemic among pregnant black women. Psychiatry Res. 2020;293:113475. Doi: 10.1016/j.psychres.2020.113475

8 Horta BL, Silveira MF, Barros AJD, Hartwig FP, Dias MS, Menezes AMB, et al. COVID-19 and outpatient care: a nationwide household survey. Cad Saude Publica. 2022;38(04):e00194121. Doi: 10.1590/0102-311 × 00194121

9 Caetano R, Silva AB, Guedes ACCM, Paiva CCN, Ribeiro GR, et al. Challenges and opportunities for telehealth during the COVID-19 pandemic: ideas on spaces and initiatives in the Brazilian context. Cad Saude Publica. 2020;36(05):e00088920. Doi: 10.1590/0102-311 × 00088920

10 Ministério da Saúde. Secretaria de Vigilância em Saúde. Departamento de Análise de Situação de Saúde (MS/SVS/DASIS) Nascidos vivos por ano e adequação quantitativa do pré-natal, por região do Brasil. Sistema de informações sobre Nascidos Vivos (SINASC) [Internet]. 2022 [cited 2022 Jul 4]. Available from: http://tabnet.datasus.gov.br/cgi/deftohtm.exe?sinasc/cnv/nvuf.def

## References

[1] Martin M M Knobel R Nandi V Pereira J G Trapani Junior A Andreucci C B Rev. Bras. Ginecol. Obstet. 2022 44 398, 10.1055/s-0041-1741450 35176779 10.1055/s-0041-1741450 PMC9948289

[2] Morón-Duarte L S Varela A R Bertoldi A D Domingues M R Wehrmeister F C Silveira M F BMC Health Serv. Res. 2021 21 1070, 10.1186/s12913-021-07053-4 34627235 10.1186/s12913-021-07053-4 PMC8501641

[3] Ministério da Saúde, Secretaria de Vigilância em SaúdeCoordenação Geral de Informações e Análises Epidemiológicas - Sistema de Informações sobre Mortalidade – SIM. Óbitos maternos por ano, por região do Brasil [Internet]. 2022 [cited 2022 Jun 16]. Available from:http://tabnet.datasus.gov.br/cgi/tabcgi.exe?sim/cnv/mat10uf.def

[4] Ministério da Saúde, Secretaria de Vigilância em SaúdeDepartamento de Análise de Situação de Saúde (MS/SVS/DASIS). Nascidos vivos por ano e adequação quantitativa do pré-natal, por região do Brasil. Sistema de informações sobre Nascidos Vivos (SINASC) [Internet]. 2022 [cited 2022 Jun 16]. Available from:http://tabnet.datasus.gov.br/cgi/deftohtm.exe?sinasc/cnv/nvuf.def

[5] Vaichulonis C G Silva R R Pinto A I Cruz I R Mazzetti A C Haritsch L Avaliação da assistência pré-natal segundo indicadores do Programa de Humanização no Pré-natal e Nascimento Rev Bras Mater Infant 2021 21 02 451 50. 10.1590/1806-93042021000200006

[6] Kotelchuck M Am. J. Public Health. 1994 84 1414; 10.2105/ajph.84.9.1414 8092364 10.2105/ajph.84.9.1414 PMC1615177

[7] Tomasi E Fernandes P A Fischer T Siqueira F C Silveira D S Thumé E Duro S M Saes M O Nunes B P Fassa A G Facchini L A Cad. Saude Publica. 2017 33 e00195815; doi: 10.1590/0102-311 × 00195815 28380149 10.1590/0102-311X00195815

[8] Ministério da Saúde Secretaria de Atenção à SaúdeDepartamento de Atenção Básica. Atenção ao pré-natal de baixo risco.Brasília (DF) Ministério da Saúde 2012

[9] Cruz G C Ruiz P C Ribeiro Junior O C Sousa A D Pereira R M Barroso C O Métodos de avaliação da qualidade de assistência ao pré-natal no Brasil: revisão integrativa da literatura Rev Eletrônica Acervo Saúde 2019 27 e521. 10.25248/reas.e521.2019

[10] Leal M C Esteves-Pereira A P Viellas E F Domingues R M Gama S G Rev. Saude Publica. 2020 54 8; 10.11606/s1518-8787.202005400145 31967277 10.11606/s1518-8787.2020054001458 PMC6961968

[11] Pedraza D F Gomes A A Rev. Cienc. Salud. 2021 19 55; 10.12804/revistas.urosario.edu.co/revsalud/a.10600

[12] Pilau G M Volpato L K Nunes R D Arq Catarin Med. 2014 43 9

